# Suppression of gain-of-function mutant p53 with metabolic inhibitors reduces tumor growth *in vivo*

**DOI:** 10.18632/oncotarget.12758

**Published:** 2016-10-19

**Authors:** Chae Lim Jung, Hyemin Mun, Se-Young Jo, Ju-Hee Oh, ChuHee Lee, Eun-Kyung Choi, Se Jin Jang, Young-Ah Suh

**Affiliations:** ^1^ Institute for Innovative Cancer Research, Asan Institute for Life Science, University of Ulsan College of Medicine, Seoul 05505, Republic of Korea; ^2^ Department of Medicinal Oncology, University of Ulsan College of Medicine, Seoul 05505, Republic of Korea; ^3^ Department of Pathology, Asan Medical Center, University of Ulsan College of Medicine, Seoul 05505, Republic of Korea; ^4^ Department of Biochemistry and Molecular Biology, School of Medicine, Yeungnam University, Daegu 42415, Republic of Korea

**Keywords:** p53 mutant knock-in mouse, gain-of-function mutation, cancer metabolism, oncogene addiction, AMPK signaling

## Abstract

Mutation of p53 occasionally results in a gain of function, which promotes tumor growth. We asked whether destabilizing the gain-of-function protein would kill tumor cells. Downregulation of the gene reduced cell proliferation in p53-mutant cells, but not in p53-null cells, indicating that the former depended on the mutant protein for survival. Moreover, phenformin and 2-deoxyglucose suppressed cell growth and simultaneously destabilized mutant p53. The AMPK pathway, MAPK pathway, chaperone proteins and ubiquitination all contributed to this process. Interestingly, phenformin and 2-deoxyglucose also reduced tumor growth in syngeneic mice harboring the p53 mutation. Thus, destabilizing mutant p53 protein in order to kill cells exhibiting “oncogene addiction” could be a promising strategy for combatting p53 mutant tumors.

## INTRODUCTION

The importance of the tumor suppressor p53 is revealed by its frequent alteration in almost all types of cancer. The majority of p53 mutations are missense and occur in the DNA-binding domain, causing a gain of oncogenic function. The mutant p53 protein fails to activate the expression of the negative feed-back regulator mdm2, and thus becomes more stable than the wild-type protein. Gains of function related to p53 mutation include enhanced cell growth, tumor metastasis and faster tumor progression [1–3].

Altered energy metabolism is a hallmark of almost all types of tumors. Neoplastic cells rapaciously take up nutrients to sustain their survival and their adaptation to metabolic stress [4, 5]. AMP-activated protein kinase (AMPK) is one of the central regulators of cellular metabolism [6], and functions as a cellular sensor of AMP levels [7]. AMPK is activated via phosphorylation on Thr-172 by upstream kinases under conditions of energetic stress [8]. Activated AMPK maintains energy homeostasis in mammalian cells through multiple pathways, and thus inhibits protein translation [9, 10], inhibits lipid synthesis [11], and regulates macroautophagy in certain circumstance [12, 13].

Diverse functions of p53 in cancer metabolism have been reported, depending on the status of this protein. Under conditions of metabolic stress, p53 is activated and induces the TP53-inducible glycolysis and apoptosis regular (TIGAR) and sestrins [14]. AMPK is reported to activate p53 tumor suppressor [15]; however, the metabolic inhibitor, metformin, activates AMPK and delays tumor progression even in the absence of p53 [16, 17]. Recently, a gain-of-function (GOF) mutant p53 was reported to negatively regulate AMPK signaling by binding to the AMPKα subunit in head and neck cancer cells. This interaction prevented the phosphorylation and activation of AMPK by liver kinase B1(LKB1), and therefore increased aerobic glycolysis and invasive cell growth [18]. Thus, the exact contributions of both wild-type and oncogenic mutant p53 to AMPK signaling need to be uncovered.

Tumors harboring mutant p53 grow rapidly with greater access to nutrients to fuel survival signaling pathways, which may render cancer cells with p53 mutations more sensitive to metabolic stress [19]. In this regard, mutant p53 could promote ‘oncogene addiction,’ the phenomenon in which cancer cell survival depends on a few oncogenes that maintain malignancy. Here, we generated a mutant p53 oncogene addiction model using tumor cells derived from mutant p53 knock-in mice, and sought to reduce tumor growth by downregulating mutant p53 expression and medicinally activating AMPK signaling.

## RESULTS

### Knockdown of GOF mutant p53 protein reduced cell proliferation

**Figure 1:**
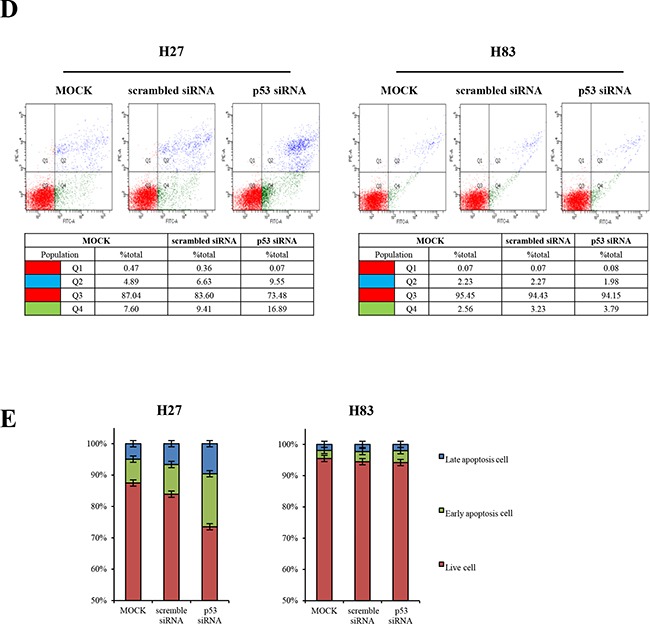
Knockdown analysis of p53 in tumor cells from GOF mutant or null mice **A.** Mouse primary cells harboring GOF mutant *p53^R172H^* (H27) or *p53^−/−^* (H83) were treated with lipofectamine 2000 (L), or transfected with 25 mM scrambled siRNA (S) or siRNA against p53 (P) for 12-48 hours. Then, mRNA and protein levels were analyzed by RT-PCR and Western blotting, respectively. After siRNA transfection, apoptosis, cell cycle arrest and signaling pathways were analyzed based on PARP cleavage, the reduction of cyclin D3 expression, and the changes of phosphorylation on AMPK and ERK, respectively, in immunoblot analysis. **B.** The growth of H27 and H83 cells was measured by the MTT assay after 48-hour treatment with lipofectamine 2000 (L), or 25 mM scrambled siRNA (S) or siRNA against p53 (P). **C.** The effects on cell growth were observed under the light microscope after 48-hour treatment with lipofectamine 2000, 25 mM scrambled siRNA or siRNA against p53. **D.** H27 cells undergoing apoptosis were analyzed by FACS with Annexin V/PI staining. Both early apoptotic (Annexin V-positive, PI-negative, Q4) and late apoptotic (Annexin V-positive and PI-positive, Q2) cells were included in cell death determinations. **E.** The results of FACS analysis are depicted as a graph, in which live cells are compared to early or late apoptotic cells.

GOF mutant p53 protein is stabilized in tumors, and has the major functions of promoting tumorigenesis and metastasis [1, 20]. The GOF p53 mutation differs from the p53 null mutation in its effects on metastasis and various tumor spectra. Tumor cells from genetically engineered mice can be used to examine the effects of a single molecular alteration (such as a missense mutation in p53) on tumorigenesis. Thus, to explore the involvement of the GOF mutant protein in oncogene addiction, we first generated different genotypes of mouse primary cancer cells from mouse tumors harboring *p53^R172H^* (H27, H36), p53-null (H83), or *p53^R172H^*/K-ras*^G12D^* mutations (H22, HL2). We at first transfected H27 and H83 cells with siRNA against p53. p53 siRNA efficiently downregulated mRNA (36-hour transfection) and protein (48-hour transfection) expression in H27 cells (Figure 1A), while scrambled siRNA did not. The knockdown of mutant p53 protein induced apoptosis and cell cycle arrest in H27 cells, as evidenced by PARP cleavage and reduced cyclin D3 expression (Figure 1A). The levels of phosphor-ERK and ERK1/2 were simultaneously decreased, but phosphor-AMPK was not reduced upon downregulation of mutant p53 protein in H27 cells. Interestingly, when cell numbers were counted after gene knockdown, cell proliferation was found to be inhibited in H27 cells (Figure 1B and 1C). When cells were analyzed by FACS with Annexin V/PI staining, approximately 35% of p53-siRNA-treated H27 cells were in the course of apoptosis (Figure 1D and 1E). Surprisingly, parallel transfection of H83 cells with siRNA-p53 had no effect on apoptosis, cell cycle arrest, or cell proliferation (Figure 1). These results critically indicate that cells expressing the GOF mutant p53 protein (H27) are addicted to this protein and depend on it to survive, while p53-null cells (H83) do not, providing evidence of a strategic way to combat p53 mutant tumors.

### Metabolic inhibitors reduced the growth of cells harboring p53 GOF alterations, and inhibited cell migration

In an effort to discover reagents that could degrade mutant p53 protein and thus impede the growth of cells addicted to this protein, we performed drug treatments on mouse tumor cells of different genotypes. Because AMPK is known to bind to p53 mutant proteins but to be released after phosphorylation and activation [18], we hypothesized that AMPK activators would induce free mutant p53. Additionally and importantly, metabolic stress can evoke chaperone-mediated autophagy (in which HSC70 guides proteins to the lysosome) instead of ubiquitin-associated degradation of mutant p53 protein [21]. Thus, we tested whether the AMPK activator phenformin, together with glucose derivative 2-DG, would induce metabolic stress and destabilize the mutant protein.

**Figure 2:**
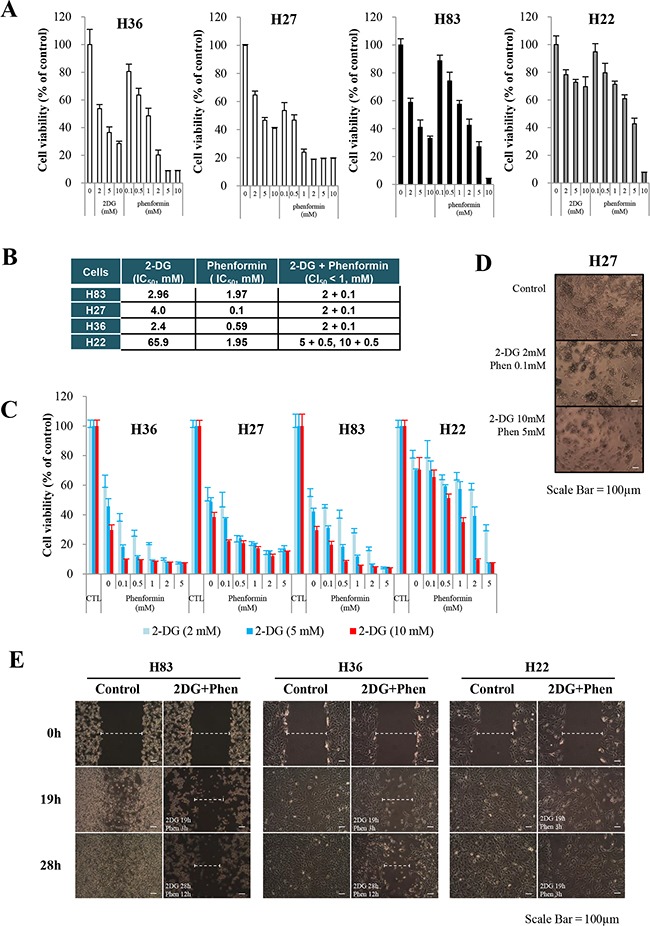
Effects of metabolic inhibitors on the growth of cells generated from p53 mutant mice Cells were treated with increasing concentrations of 2-DG and/or phenformin for 24 hours. Viability was determined by the MTT assay. The absorbance was measured with an ELISA plate reader (Molecular Devices), and the inhibitory effects were normalized to untreated conditions. **A.** Growth graphs are depicted for mouse primary cells harboring GOF mutant *p53^R172H^* (H27, H36), *p53^null^* mutant (H83), or *p53^R172H^/K-ras^LAI^* (H22), exposed to increasing concentrations of each drug (up to 10 mM). **B.** Effects of 2-DG and phenformin alone and in combination on various p53-mutant cells, based on comparison of the IC_50_ and CI values. **C.** Growth graphs were created for primary H27, H36, H83, or H22 cells treated with different concentrations of the two drugs (phenformin, 0.1-5 mM; 2-DG, 2-10 mM). As an evaluation of the combinational effect of the two drugs, cells were pretreated with 2-DG for 8 hours, followed by further treatment with both drugs for 15 hours. Scale bar in the photograph corresponds to 100 μm. **D.** Retarded growth of H27 cells was observed under a light microscope after cells were treated with 2-DG and phenformin. Scale bar in the photograph corresponds to 100 μm. **E.** Plates with confluent layers of H83, H36 and H22 cells were scratched and monitored for up to 28 hours in a wound healing assay. **F.** The effects of metabolic inhibitors on the growth of human NSCLC cells (A549, H23) were analyzed with increasing concentrations of 2-DG and/or phenformin for 24 hours. Viability was determined by the MTT assay. **G.** The activation of ERK and AMPK was analyzed after A549 and H23 cells were treated with 2-DG and phenformin.

Cells were incubated with varying concentrations of 2-DG or phenformin for 24 hours. Individually, each drug inhibited cell growth in all the cells tested; H27, H36 and H83 cells were highly sensitive to the treatments, while H22 cells exhibited higher IC_50_ values than the other cells (Figure 2A and 2B). Following treatment with a combination of both reagents, the growth of H27, H36 and H83 cells was severely impaired, as the combination index 50 (CI_50_) of less than 1 was the treatment of 2 mM 2-DG plus 0.1 mM phenformin, while the growth of H22 cells was less inhibited, as CI_50_ of less than 1 was the treatment of 5 to 10 mM 2-DG plus 0.5 mM phenformin (Figure 2B and 2C). Morphological cell death was obvious after 24 hours of treatment (representative microscopic pictures are shown in Figure 2D). In a wound healing assay to test the inhibitory effects of the drugs on cell migration, the gaps created on the H83 and H36 cell plates were only 50% and 60% covered, respectively, while H22 cells migrated and filled more than 90% of the gap after 28 hours (Figure 2E), indicating that the metabolic inhibitors adversely affected the migration of p53-mutant cells, including p53-null cells, although they were less harmful to cells containing both p53- and K-ras mutations. These results may suggest that K-ras mutation promotes resistance to these drugs by activating complex survival signaling pathways.

The antitumor effects of these drugs were also observed in the human NSCLC cell lines, A549 and H23, containing wild-type and mutant p53, respectively (Figure 2F). Interestingly, the proliferation of A549 cells was less inhibited than that of H23 cells. The pattern of p53 expression differed between these two cell lines following drug treatment; p53 protein was degraded in H23 cells, but its expression increased in A549 cells, although the dephosphorylation of ERK was observed in both cell lines. The phosphorylation of AMPK was affected differently in the two cell lines, which was not induced in A549 cells but increased shortly in H23 cells (Figure 2G). These results may indicate that drugs have different modes of action, depending on the status of p53.

### Metabolism inhibitors suppressed the growth of cells harboring p53 alterations

**Figure 3:**
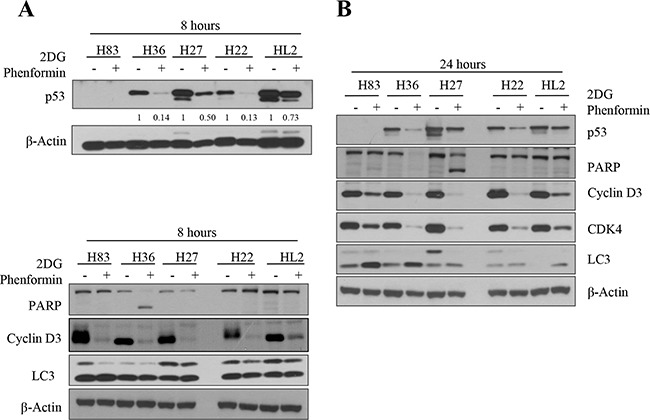
Protein expression was analyzed after p53 mutant cells were treated with 2-DG and phenformin **A.** Mouse primary cells of different p53 genotypes (*p53^−/−^*: H83, *p53^R172H^*: H27 and H36, *p53^R172H^/K-ras^G12D^*: H22, HL2) were incubated with 2 mM 2-DG and 1 mM phenformin for 8 hours. Whole-cell lysates (30 μg) were analyzed by Western blotting with antibodies against p53, PARP, cyclin D3, LC3, and β-actin. **B.** Cells were incubated with 2 mM 2-DG and 1 mM phenformin for 24 hours, followed by Western blotting with antibodies against p53, PARP, cyclin D3, CDK4, LC3, and β-actin. **C.** H27 and H83 cells undergoing apoptosis were analyzed by FACS with Annexin V/PI staining after drug treatment. Both early apoptotic (Annexin V-positive, PI-negative, Q4) and late apoptotic (Annexin V-positive and PI-positive, Q2) cells were included in cell death determinations. **D.** The results of FACS analysis are depicted as a graph, in which live cells are compared to early or late apoptosis cells. **E.** The effects of 2-DG and phenformin on the AMPK pathway in p53 mutant cells were evaluated. Mouse primary cells of various *p53* genotypes (H83, H27, H36, H22, HL2) were treated with 2-DG and phenformin for 8 or 24 hours, and the levels of phospho-AMPK and phospho-p70S6K (S371), together with the total level of each protein, were analyzed by Western blotting.

As the stabilized mutant p53 protein promotes tumor cell growth ([22] and our gene knockdown experiments in Figure 1), we analyzed its expression after cells were treated with metabolic inhibitors. The levels of mutant p53 protein were reduced in H36 and H27 cells, and to a lesser extent in H22 and HL2 cells, after treatment with phenformin and 2-DG for 8 hours (Figure 3A) and 24 hours (Figure 3B). The treatment of H36 and H27 cells with these drugs induced PARP cleavage and reduced cyclin D3 expression, indicating that mutant tumor cells underwent apoptosis and cell cycle arrest, which might have synergistically retarded cell growth. These effects were also observed in H83 cells lacking p53 expression during the same time period. Although reduced cyclin D3 and CDK4 expression were also observed in H22 and HL2 cells, PARP cleavage was not observed until 24 hours of treatment (Figure 3B).

The anti-proliferative mechanism was further examined through FACS analysis. About 45% of H27 cells and 25% of H83 cells underwent apoptosis when treated with 2mM 2-DG plus 1 mM of phenformin (Figure 3C, D). Phenformin-only treatment reduced p53 expression, activated the AMPK pathway, and promoted caspase 3 cleavage and cyclin D3 degradation in p53-mutant cells (Supplementary Figure S1). These results suggest that inhibition of tumor cell metabolism blocks cell proliferation.

### Drug treatment inhibited the AMPK signaling pathway

Biguanides induce the phosphorylation of the stress-sensitive kinase AMPK, followed by the regulation of pivotal metabolic pathways, such as hyperactivation of 4E-binding protein1 [23]. To validate the involvement of AMPK signaling in the growth inhibition of mutant cells, we analyzed the expression of proteins in this signaling pathway. As shown in Figure 3E, phospho-AMPK was observed in mouse tumor cells after 8 hours of treatment with the drugs. This was accompanied by alterations in the mTOR signaling pathway, such as dephosphorylation of p70S6k at S371.

**Figure 4:**
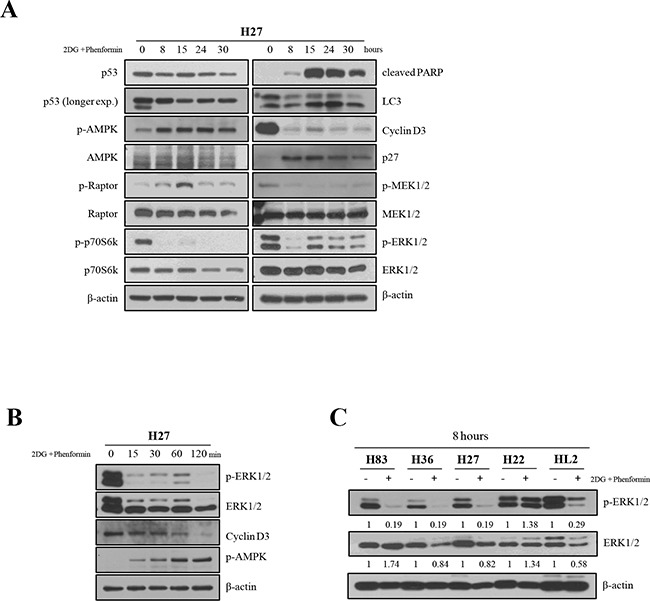
The effects of 2-DG and phenformin on AMPK signaling were examined in H27 cells over a time course **A.** The expression of p53, phospho-AMPK, phospho-raptor and phospho-p70S6K (S371), as well as the total level of each protein, were analyzed by Western blotting. Apoptosis (cleaved PARP; C-PARP), cell cycle arrest (cyclin D3 and p27) and autophagy (LC-3) in H27 cells upon drug treatment were also examined by Western blotting. The MAPK signaling pathway was examined in H27 cells by Western blotting with antibodies for phospho-MEK1/2, MEK1/2, phospho-ERK and ERK. **B.** The effects of 2-DG and phenformin on H27 cells at early time points were examined based on phospho-ERK, ERK, cyclin D3, and phospho-AMPK levels. **C.** The ERK of the MAPK signaling pathway was further investigated in various cell types (H83, H27, H36, H22, HL2) by Western blotting with antibodies for phospho-ERK and ERK after 8 hours of treatment.

The effects of the drugs on signaling pathways in H27 cells were further examined over the course of time (Figure 4A). In the AMPK pathway, phospho-raptor appeared after 15 hours of treatment, and, interestingly, dephosphorylation of p70S6k (S371) occurred within 8 hours. These results indicate that the activation of AMPK signaling may inhibit protein and lipid synthesis by interfering the mTOR pathway. Apoptotic and autophagic events became clear after 15 hours, and alterations typical of cell cycle arrest were observed after 8 hours, such as the reduction of cyclin D3 expression and the activation of p27. The levels of phospho-ERK and phospho–MEK1/2 were dramatically reduced after 8 hours of treatment, while the total protein amounts were unchanged, and these effects were maintained up to 24 hours of treatment.

When H27 cells were then examined at early time points of treatment (Figure 4B), phosphorylated ERK was barely detected at 15 minutes. Interestingly, phospho-AMPK levels correlated inversely with cyclin D3 levels upon treatment, as phospho-AMPK expression gradually increased after 15 minutes and cyclin D3 disappeared after 2 hours. Obvious dephosphorylation of ERK was detected in H83, H36 and H27 cells within 8 hours of treatment with 2-DG and phenformin, although the total ERK level decreased slightly in H36 and H27 cells. Dephosphorylation of ERK, however, was not detected in H22 cells or was less in HL2 cells at the 8-hour time point, suggesting that ERK signaling pathways may be highly activated in these cells (Figure 4C). These results indicate that the growth of mutant cells may be inhibited as mutant p53 protein is degraded, and that multiple cell survival signaling pathways may be inhibited by these metabolism inhibitors.

### AMP increase and HSP70/90 chaperones were involved in the destabilization of mutant p53

**Figure 5:**
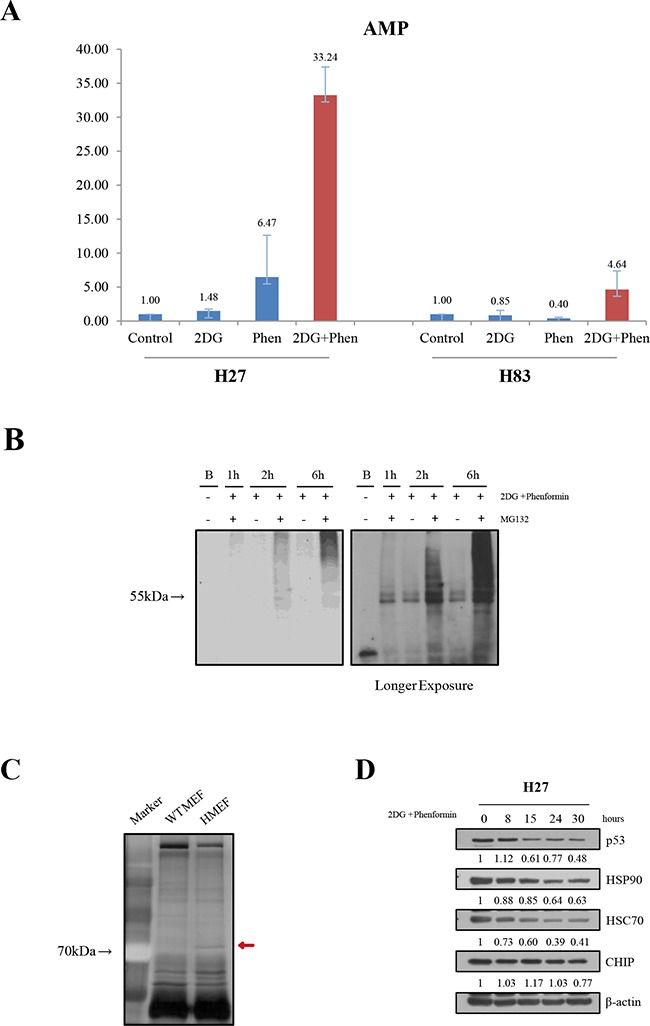
Mechanistic exploration of the cell growth retardation upon treatment with metabolic inhibitors **A.** The change of adenosine was analyzed in H27 and H83 cells. After drug treatment, cell lysates were subjected to LC-MS/MS, and the relative amounts of AMP were determined. **B.** Ubiquitination analysis was performed in the presence of a proteasome inhibitor in H27 cells upon drug treatment. Cell lysates treated with 2-DG and phenformin and/or MG132 for different lengths of time were immunoprecipitated with an anti-p53 antibody (PAb240) and Protein G-Sepharose 4 Fast Flow beads overnight at 4°C, and blotted with an anti-ubiquitin antibody. **C.** Mutant p53 binding partners were analyzed. Cell lysates of wild-type MEFs (wt MEF) or *p53^R172H^* MEFs (HMEF) were immunoprecipitated with an anti-p53 antibody (FL393) and separated on a sodium dodecyl sulfate polyacrylamide gel, followed by visualization of staining. The red arrow indicates a unique band of approximately 70 kDa detected in HMEF cells, followed by further analysis by mass spectrometry. **D.** The expression of p53, HSP90, HSC70, and CHIP were evaluated by Western blotting over the time course of drug treatment.

The mechanisms regulating mutant p53 protein stability have been intensively explored [20–22, 24]. Mutant p53 can be inhibited by mdm2 E3 ligase, although this ligase cannot be induced in tumors harboring the mutant p53 that is incompetent as a transcription factor. Also, chaperones help to stabilize mutant p53 proteins. Mutant p53 is degraded through chaperone-mediated autophagy in glucose-free conditions. To explore the critical molecules involved in mutant protein instability upon drug treatment, we first analyzed the changes in adenosine phosphate levels. When the levels of AMP, ADP and ATP were analyzed, a clear loss of ATP (data not shown) and AMP increase were observed in H27 cells following treatment with phenformin, either alone or combined with 2-DG. However, there was little change in H27 cells treated with 2-DG alone. Interestingly, the drugs had the least effect on H83 cells, indicating that the increase of AMP is unique to p53 mutant cells (Figure 5A). We also examined the ubiquitination of mutant p53 in H27 cells upon drug treatment. When cells were treated with the drugs together with the proteosome inhibitor MG132, higher-molecular-weight proteins that had immunoprecipitated with the p53 antibody were detected with ubiquitin in H27 cells, indicating that proteosomal degradation was responsible for the destabilization of mutant p53 upon drug treatment (Figure 5B).

Protein-protein interactions were also examined through immunoprecipitation with the p53 antibody in wild-type or mutant p53 MEFs. A unique band around 70 kDa in mutant MEFs was analyzed by mass spectrometry (Figure 5C and Table 1). The expression of heat-shock cognate protein 70 (HSC70) and its family member heat shock protein 90 (HSP90) were further analyzed in H27 tumor cells upon drug treatment. HSC70 and HSP90 levels were reduced after drug treatment, in parallel with the reduction in mutant p53 expression. The expression of E3 ligase C-terminal HSC70 interacting protein (CHIP) [25], however, was not reduced by the metabolism inhibitors (Figure 5D). These results suggest that the mutant protein is destabilized by multiple mechanisms, and that the E3 ligase MDM2, together with chaperones, is critical for the stabilization of mutant p53.

**Table 1:** Binding partners of mutant p53 identified through peptide mass spectrometry analysis of proteins immunoprecipitated with a p53 antibody

No.	Accession	Reference [Mus musculus]	Score^a^	MW^b^	Peptide
1	31981690	heat shock cognate 71-kDa protein	220.24	70827.3	31
2	163310765	serum albumin precursor	160.29	68647.8	34
3	70995287	calcineurin binding protein 1	70.20	243016.2	8
4	112293264	protein disulfide-isomerase A3 precursor	40.14	56642.8	4
5	31980648	ATP synthase subunit beta, mitochondrial precursor	30.21	56265.6	3
6	183396771	60-kDa heat shock protein, mitochondrial	30.20	60917.5	3
7	31982755	vimentin	30.19	53655.2	3
8	306482623	histone H4	30.18	11360.4	3
9	162461907	stress-70 protein, mitochondrial	30.16	73415.7	3
10	6671509	actin, cytoplasmic 1	20.21	41709.7	5
11	20330802	serotransferrin precursor	20.21	76673.8	2
12	30425250	beta-actin-like protein 2	20.20	41977.0	2
13	84781771	trypsin 10 precursor	20.19	26203.9	7
14	124339826	heat shock 70-kDa protein 1B	20.18	70133.2	8
15	254540168	78-kDa glucose-regulated protein precursor	20.16	72377.6	2
16	6755863	endoplasmin precursor	20.15	92418.1	2
17	357588427	alpha-1-antitrypsin 1-1 isoform 2	20.15	48765.2	2
18	6671507	actin, aortic smooth muscle	20.15	41981.8	2
19	31982186	malate dehydrogenase, mitochondrial precursor	10.26	35588.8	1
20	124339838	heat shock 70-kDa protein 1-like	10.25	70593.3	3

a Proteins with a score higher than 10 are listed.

b Daltons

### Treatment with 2-DG and phenformin induced tumor regression in a syngeneic p53 mutant mouse model

**Figure 6:**
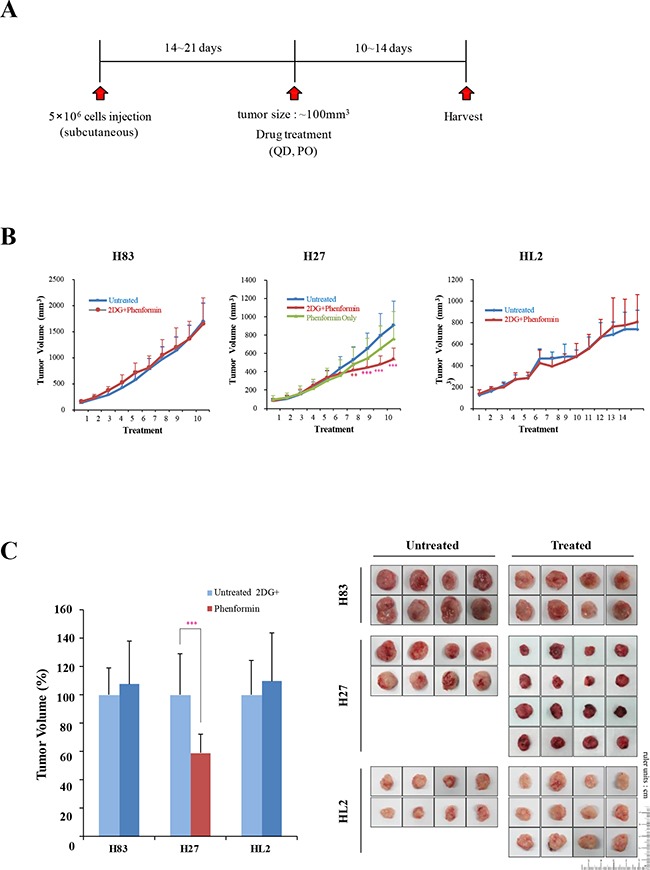
The effects of 2-DG and phenformin on cell migration and tumor growth were explored with p53 mutant cell grafts **A.** *In vivo* experimental schemes are summarized in the time course. H83, H27 and HL2 cells formed tumors of approximately 100 mm^3^ within 14-21 days of inoculation. Mice were then treated orally with PBS (denoted as ‘not treated’) or 250 mg/kg phenformin and 2-DG (denoted as ‘2-DG + phenformin’) once per day for 10-14 days. **B.** The tumor volume was calculated daily with the formula for an ellipsoid sphere: W1 × W 2 × W2/2 = x mm^3^, where W1 represents the largest tumor diameter and W2 the smallest tumor diameter. Independent xenograft experiments were performed three times. *P < 0.01, **P < 0.005 and ***P < 0.001 refer to comparisons between vehicle- and drug-treated tumors. Animal body weights were monitored during the experiment. **C.** Representative tumors from PBS- (black box, not treated) or drug-treated (white box, 2-DG + phenformin) mice were collected at the end points of the experiment (right). Growth was graphically depicted according to size (left). **D.** Western blotting analysis of p53 and PARP expression in tumors harvested from syngeneic grafts. **E.** The relative changes in p53 and cleaved PARP levels in Western blotting analysis are shown as a graph. ***P < 0.001 refers to comparisons between vehicle- and drug-treated tumors.

To explore the *in vivo* efficacy of the drugs, we performed syngeneic graft experiments by inoculating athymic male nude mice (CD-1 *nu*/*nu*) with mutant p53 tumor cells (H83, H27, or HL2 cells). When the tumor volume reached approximately 100 mm^3^, the mice were treated orally with 2-DG and phenformin, and tumor sizes were measured for 10-14 days (the experimental scheme is summarized in Figure 6A). Interestingly, tumor growth was significantly reduced in the H27-grafted mice (p < 0.001), although no difference was detected in the H83- or HL2-grafted mice (Figure 6B). The body weights of the treated mice were unchanged (Supplementary Figure S2), indicating no significant cytotoxicity of the drugs. The average tumor sizes were reduced to 50% of untreated levels in H27-cell syngeneic mice (Figure 6C). The expression of mutant p53 protein was reduced to 70% and PARP cleavage increased to 170% of control levels in tumors treated with 2-DG and phenformin (Figure 6D, E). Thus, combination treatment with 2-DG and phenformin suppressed p53 mutant tumors and induced tumor regression, mutant protein destabilization and apoptosis. These results also support the notion that K-ras mutation promotes resistance to these drugs by activating complex survival signaling pathways, such as ERK phosphorylation.

## DISCUSSION

*TP53* tumor suppressor is mutated in almost all cancer types, and the proteins encoded by mutated forms of the gene are occasionally more stable than the wild-type protein, endowing tumor cells with gains of function [20]. Thus, there has been considerable interest in developing therapeutic strategies that re-activate the p53 pathway or suppress oncogenic mutated p53 to halt cancer progression [26, 27]. Cancer therapeutics for mutated p53 have been developed previously, such as Prima-1^MET^, which converts p53 from a misfolded protein conformation to its wild-type form [28, 29]. Although the effects of such therapies on tumor cell growth have been tested in clinical trials, their efficacy in cancer therapy remains unclear.

**Figure 7:**
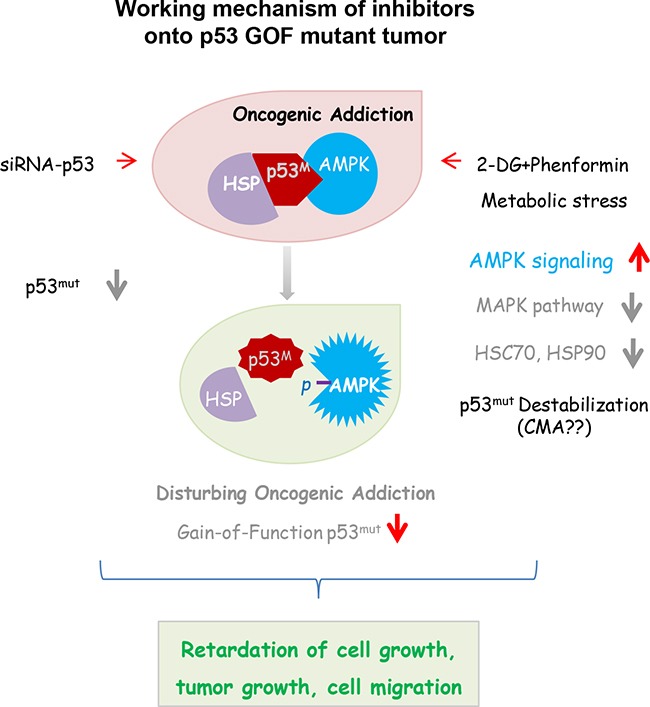
Synergistic influence is expected in cells harboring a GOF p53 mutation, when oncogene addiction and tumor metabolism are simultaneously disturbed

In this study, we found that tumor cells harboring mutant *TP53* depended on this GOF protein to survive, and that destabilization of this protein reduced tumor growth *in vivo*. When the expression of mutated p53 was abolished through gene-specific downregulation, the growth of tumor cells with the GOF mutation was decreased, indicating that cells were addicted to the conformational disordered p53 protein. Combinatorial treatment of cells with two metabolic inhibitors, 2-DG and phenformin, caused metabolic stress and the degradation of the mutant p53 protein. The growth of tumors bearing p53 mutations was also inhibited by treatment with these two reagents *in vitro* and *in vivo* through multiple mechanisms, implying that mutant p53 tumors are oncogene-addicted. The working model based on these results is depicted in Figure 7, wherein metabolism inhibitors are shown orchestrating multiple growth inhibitory cascades in tumor cells addicted to mutant p53. Importantly, treatment strategies that take advantage of the dependency of some tumors on GOF mutant p53 could be beneficial, as which is utilized for suppressing EGFR or other oncogenes [30–32]. There is increasing evidence that stabilized oncogenic mutant p53 protein can be exploited as a cancer-specific drug target [33, 34]. Notably, this study demonstrated that metabolic inhibitors also challenge mutant p53 dependency and serve as cancer therapeutics.

Biguanide compounds have recently been rediscovered, due to their anticancer efficacy. In particular, metformin has been studied in multiple clinical trials [16, 35]. Phenformin is also able to inhibit tumor cell growth in liver kinase B1-mutated cancer cells. However, 2-DG was recently shown not only to enhance the inhibition of cell growth, but also to reverse the acidification caused by phenformin treatment in colon cancer cells [36], providing beneficial evidence for the development of phenformin as a promising cancer therapeutic. In this regard, our current results are in agreement with the repositioning of phenformin as an anticancer drug.

There is a report that AMPK-HSC70 axis promotes degradation of fat-specific protein 27 (FSP27), which performs critical functions in energy metabolism, through the proteasomal ubiquitin-dependent protein catabolic process. [37]. The GOF mutant p53 is known to bind to the HSC70 chaperone for further processing [25, 38]. In our mass spectrometry-based proteomic analysis, the mutant p53 protein bound tightly to HSC70, and HSC70 was negatively regulated by 2-DG and phenformin, indicating that the AMPK-HSC70 axis is a key pathway regulating the stability of mutant p53 protein.

Although wild-type p53 is mainly regulated by MDM2, its mutant form is reported to be more sensitive to chaperone-mediated autophagy, especially in metabolic stress conditions [21]. The mechanism whereby metabolic inhibitors retard mutant cell growth may be chaperone-mediated autophagy, in which drugs accelerate the existing metabolic stress on cells with high energy demands by reducing glucose levels and increasing AMP levels.

One of the main causes of tumor initiation and maintenance is the acquisition of gene mutations on driver oncogenes/tumor suppressors. Although extensive cancer genome analysis and functional studies have revealed that established human cancers harbor, on average, 30-60 mutations capable of altering protein functions, compelling data from preclinical and clinical trials indicate that many cancers are sensitive to therapeutics that inhibit a single oncogene. These results support the concept of oncogene addiction, whereby cancer cell survival depends on a few oncogenes that maintain malignancy. Indeed, the oncogenic mutant p53 was shown to inhibit AMPK activity, promoting cell growth and cancer cell metabolism [18]. In our current study, phenformin together with 2-DG efficiently induced the phosphorylation of AMPK in p53 tumor cells, destabilized mutant p53 protein, and inhibited cell growth. In ongoing preclinical and molecular studies, we will seek to validate the tumor growth inhibitory effects of metabolic inhibitors as potential cancer therapeutics.

## MATERIALS AND METHODS

### Mice, mouse cancer cells, chemicals, and reagents

Genetically engineered *p53^R172H^* knock-in mice and *K-ras^G12D^* transgenic mice were kindly provided by Dr. Lozano (MD Anderson Cancer Center, University of Texas, Houston, TX), and by the National Cancer Institute (NCI, Frederick, MD), respectively. *p53^+/−^*mice were purchased from The Jackson Laboratory (Bar Harbor, ME). Mouse primary cancer cells were obtained from tumors harboring *p53^R172H^* (H27, H36), *p53^−/−^*(H83), or *p53^R172H^*/K-ras*^G12D^* (HL2, H22). The tumors of H27 and H36 were sarcomas, the tumor of H83 was rhabdomyosarcoma, and the tumors of HL2 and H22 were adenocarcinomas. Cells grown from tumor fragments were incubated in DMEM supplemented with 10% fetal bovine serum, 2 mM l-glutamine, 100 IU/mL penicillin and 100 μg/mL streptomycin (Invitrogen, Carlsbad, CA) in 5% CO_2_. Mouse embryonic fibroblasts (MEFs) were obtained from 13.5-day embryos and grown in the same medium as tumor cells. Phenformin and 2-deoxyglucose (2-DG) were purchased from Sigma Chemical Co. (St. Louis, MO). Human non-small cell lung cancer (NSCLC) cell lines, A549 and H23, were obtained from the American Type Culture Collection (Manassas, VA) and cultured in RPMI1640 supplemented with 10% fetal bovine serum, 2 mM l-glutamine, 100 IU/mL penicillin and 100 μg/mL streptomycin in 5% CO_2_.

### Cell proliferation and wound healing assays

Cell viability was determined with the MTT (3-(4, 5-Dimethylthiazol-2-yl)-2,5-Diphenyltetrazolium Bromide) assay. Cells were exposed to 2-DG and phenformin for different time intervals and then were incubated with 0.5-mg/mL MTT solution (Sigma, St. Louis, MO). The resulting formazan dye color was measured at 570 nm.

For analysis of cell invasion/migration, cells were seeded at >90% confluence, and a straight scratch was made in the middle of each dish. The cells invading across the scratch were photographed under a light microscope.

### Gene knockdown analysis and reverse transcriptase polymerase chain reaction (RT-PCR)

H27 or H83 cells at 60% confluence were transfected with 25 nM scrambled small interfering RNA (siRNA) or p53-siRNA (ON-TARGET plus SMART pool Mouse Trp53, GE Dharmacon Inc., Lafayette, CO) mixed with Dharmafect (GE Dharmacon Inc.) for 12-48 hours. Downregulation of p53 was evaluated at the mRNA and protein levels. Total RNA was extracted with Trizol reagent (Ambion, Grand Island, NY), and RT-PCR was performed with a High Capacity cDNA Reverse Transcription kit (Applied Biosystems). Primers were obtained from Bioneer (Gyungkido, Republic of Korea): forward primer 5′-ACCTGGGCTTCCTGCAGTCT-3′ and reverse primer 5′-CGCTGACCCACAACTGCAC-3′ for p53, and forward primer 5′-TCACCACCATGGAGAAGGC-3′ and reverse primer 5′-GCTAAGCAGTTGGTGGTGCA-3′ for GAPDH. To verify the gene knockdown, we further analyzed protein levels by Western blotting. A trypan blue exclusion assay was performed, and cells were counted with an Automated Cell Counter (BioRad, Hercules, CA) as a measure of cell growth suppression due to gene downregulation. Three independent experiments were performed to confirm the gene downregulation.

### Western blotting analysis

Logarithmically growing tumor cells were collected and incubated in a lysis buffer containing a mixture of protease and phosphatase inhibitors (Sigma) on ice. Protein concentrations were determined with a Protein Assay Dye Reagent Concentrate (Bio-Rad), and equal amounts were separated on sodium dodecyl sulfate-polyacrylamide gels and transferred to nitrocellulose membranes (GE Healthcare, Wauwatosa, WI). The antibodies used for Western blotting were obtained as follows: p53, β-actin, p70S6K, MAPK/ERK kinases 1 and 2(MEK)1/2, ERK, HSP90 and HSC70 from Santa Cruz (Dallas, TX); and PARP, cyclin D3, CDK4, p-AMPK, p-raptor, p-p70S6K, p-4EBP1, cleaved PARP, p27, p-MEK1/2, p-ERK and ubiquitin from Cell Signaling (Boston, MA). The intensities of the protein bands were quantified with Quantity One software.

### Ubiquitination and protein-protein interaction analysis

To analyze whether mutant p53 underwent proteasomal degradation upon drug treatment, we incubated cells with the proteasome inhibitor MG132 (Enzo Life Science, Farmingdale, NY) during drug treatment, and analyzed ubiquitination. Briefly, treated cells were lysed with lysis buffer as described in the Western blotting section, and immunoprecipitation was performed with an anti-p53 antibody (PAb240) and Protein G-Sepharose 4 Fast Flow beads (GE Healthcare) overnight at 4°C. The immunoprecipitates were washed with phosphate-buffered saline (PBS) and blotted with an anti-ubiquitin antibody.

Mutant p53-associated proteins were isolated by tandem affinity purification. After immunoprecipitation of wild-type or mutant p53 from MEF cell lysates, the bound proteins were visualized by silver staining. Unique bands found only in *p53^R172H^* mouse embryo fibroblast (MEFs), not in wild-type MEFs, were extracted and analyzed via mass spectrophotometry.

### *In vivo* experiments

Athymic male nude mice (CD-1 *nu*/*nu* from Jungang Inc., Seoul, Republic of Korea) were used for *in vivo* tumor growth studies. All animal experiments were carried out with the approval of the Institutional Experimental Animal Care and Use Committee. H27, H83 or HL2 cells were suspended at 5 × 10^6^ cells per 200 μL in PBS and injected into the legs of four-week-old mice. When the tumors were approximately 100 mm^3^ in size (10-14 days), the animals were randomly divided into three groups. Following randomization, the mice were treated with vehicle, 0.2 mL of phenformin, or 2-DG plus phenformin (750 mg/kg of 2-DG and 100 mg/kg of phenformin) orally once per day for the duration of the experiment. The numbers of mice were as follows: H83, eight mice each for the untreated and treated groups with 2-DG plus phenformin; H27, eight mice for the untreated and 16 mice for the treated group; and HL2, eight mice for the untreated and 12 mice for the treated group.

Tumor size was measured with digital calipers daily. Tumor measurements were converted to tumor volumes through the following formula: W1 × W2 × W2/2 = x mm^3^ (where W1 and W2 represent the largest and smallest tumor diameters, respectively). Mice were sacrificed when the tumors in the control group exceeded 1500 mm_3_. Tumors were excised and assessed histologically for verification of growth. Statistical significance was determined with Student's *t*-test [39].

### Flow cytometric analysis through Annexin V/propidium iodide double staining

For Annexin V/Propidium iodide (PI) assays, cells were stained with Alexa Fluor 488 Annexin V and PI, and apoptosis was evaluated by flow cytometry, according to the manufacturer's protocol (Invitrogen). Briefly, 2 × 10^5^ cells were stained with 5 μL of Annexin V-FITC and 1 μL of PI (100 μg/mL) in 1X binding buffer (50 mM HEPES, 700 mM NaCl, 12.5 mM CaCl_2_, pH 7.4) for 15 min in the dark. The number of apoptotic cells was determined with a FACSCanto II flow cytometer (BD Biosciences, San Jose, CA). Both early apoptotic (Annexin V-positive, PI-negative) and late apoptotic (Annexin V-positive and PI-positive) cells were included in cell death determinations.

### Metabolite analysis

For the metabolism assay, 1 × 10^6^ cells were lysed with 80% methanol and chloroform, and then were centrifuged at 13,000 rpm for 10 min. The aqueous layer was collected and analyzed by LC-MS/MS QTRAP (AB SCIEX, Framingham, MA, US).

### Drug combination studies

Data analysis for the additive/synergistic effects of the drugs was performed with the CompuSyn program (Chou and Martin). The resulting combination index (CI) theorem of Chou-Talalay offers quantitative definitions for additive effects (CI=1), synergism (CI<1) and antagonism (CI>1) in drug combinations.

## SUPPLEMENTARY MATERIALS FIGURES


