# Supplementary material for: Suppression of gain-of-function mutant p53 with metabolic inhibitors reduces tumor growth in vivo
Source: Oncotarget. 2016 Oct 19;7(47):77664–82. doi: 10.18632/oncotarget.12758 (PMC5363612; doi:10.18632/oncotarget.12758)
Supplement: Supplementary file 1 [file oncotarget-07-77664-s001.pdf]

Suppression of gain-of-function mutant p53 with metabolic inhibitors reduces tumor growth *in vivo*

SUPPLEMENTARY FIGURES

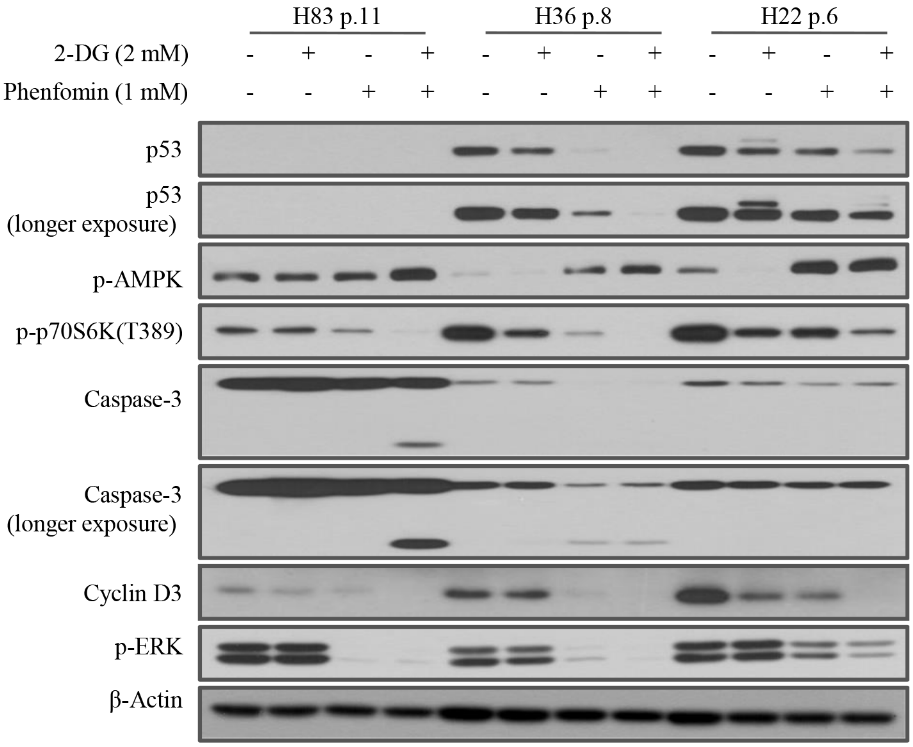

Supplementary Figure S1: Western blotting analysis of p53 stabilization and cell growth signaling factors after treatment with 2-DG and phenformin. H83, H36 and H22 cells were treated with 2 mM 2-DG and/or 1 mM phenformin for 24 hours.

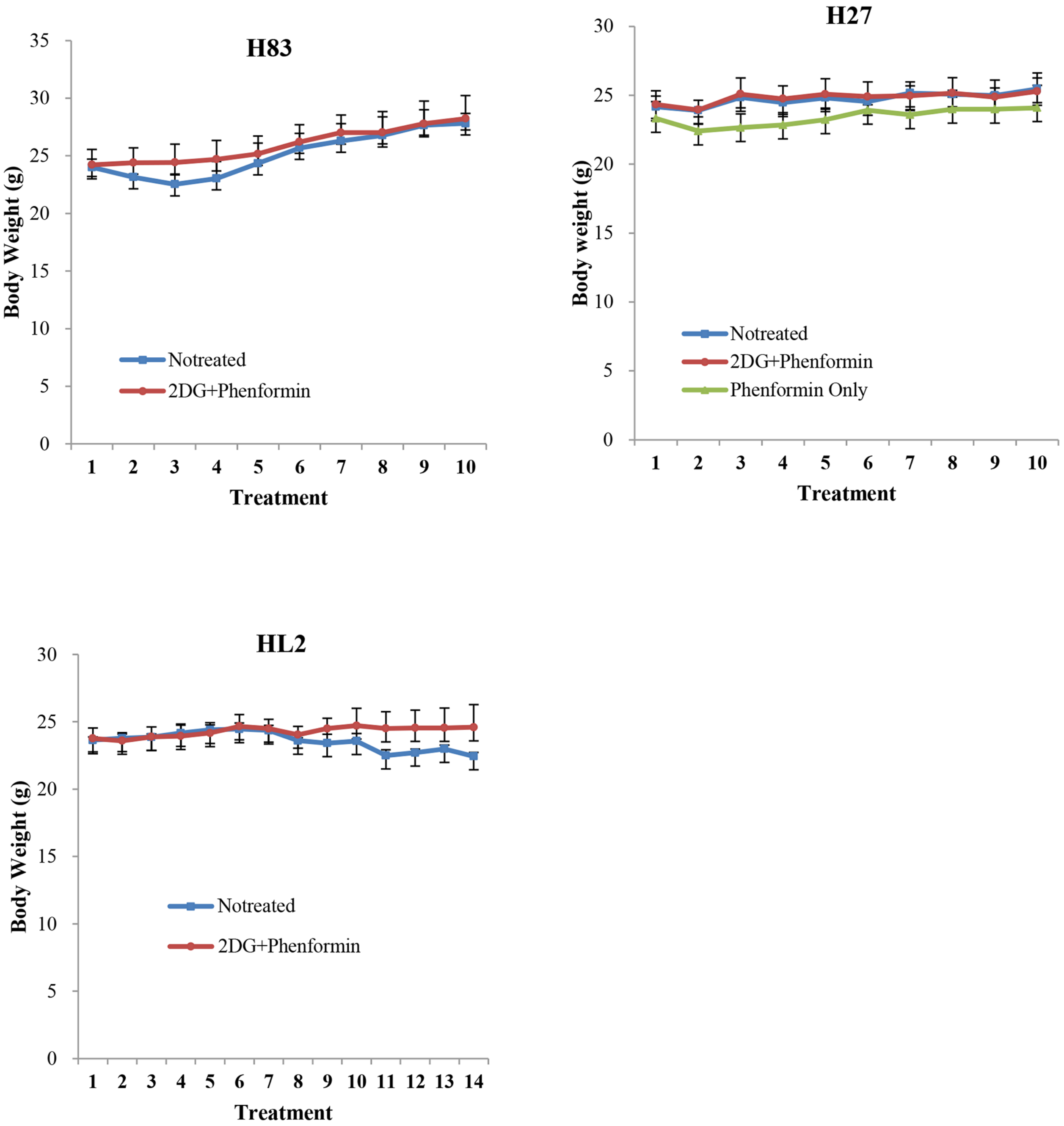

Supplementary Figure S2: Body weights of mice used to study the effects of 2-DG and phenformin on tumor growth were monitored during drug treatment.
